# A cohort study of the association between secondary sex ratio and parental exposure to polybrominated biphenyl (PBB) and polychlorinated biphenyl (PCB)

**DOI:** 10.1186/1476-069X-8-35

**Published:** 2009-08-15

**Authors:** Metrecia L Terrell, Alissa K Berzen, Chanley M Small, Lorraine L Cameron, Julie J Wirth, Michele Marcus

**Affiliations:** 1 Department of Epidemiology, Rollins School of Public Health, Emory University, 1518 Clifton Rd, Atlanta, Georgia, 30322; USA; 2 Division of Environmental Health, Michigan Department of Community Health, 201 Townsend, Lansing, Michigan, 48913; USA; 3 Department of Epidemiology, Michigan State University, East Lansing, Michigan, 48824; USA; 4 Departments of Obstetrics and Gynecology, Michigan State University, East Lansing, Michigan, 48824; USA; 5 Department of Environmental and Occupational Health, Rollins School of Public Health, Emory University, Atlanta, Georgia, 30322, USA

## Abstract

**Background:**

Polybrominated biphenyl (PBB), a brominated flame retardant, was accidently mixed into animal feed in Michigan (1973–1974) resulting in human exposure through consumption of contaminated meat, milk and eggs. Beginning in 1976 individuals who consumed contaminated products were enrolled in the Michigan Long-Term PBB Study. This cohort presents a unique opportunity to study the association between parental exposures to PBB and offspring sex ratio.

**Methods:**

We identified offspring of female PBB cohort participants (born 1975–1988) and obtained electronic birth records for those born in the state of Michigan. We linked this information to parental serum PBB and PCB concentrations collected at enrollment into the cohort. We modeled the odds of a male birth with generalized estimating equations accounting for the non-independence of siblings born to the same parents. We explored potential confounders: parental age and education at offspring's birth, parental body mass index at cohort enrollment, birth order, gestational age and year of offspring's birth.

**Results:**

The overall proportion of male offspring among 865 live births to cohort mothers was 0.542. This was higher than the national male proportion of 0.514 (binomial test: p = 0.10). When both parents were in the cohort (n = 300), we found increased odds of a male birth with combined parents' enrollment PBB exposure ≥ the median concentrations (3 μg/L for mothers; 6 μg/L for fathers) compared to combined parents' PBB exposure < the median concentrations (AOR = 1.43, 95% CI: 0.89–2.29), although this did not reach statistical significance. In addition, there was a suggestion of increased odds of a male birth for combined parents' enrollment PCB exposure ≥ the median concentrations (6 μg/L for mothers; 8 μg/L for fathers) compared to combined parents' enrollment PCB exposure < the median concentrations (AOR = 1.53, 95% CI: 0.93–2.52).

**Conclusion:**

This study adds to the body of literature on secondary sex ratio and exposure to environmental contaminants. In this population, combined parental exposure to PBBs or PCBs increased the odds of a male birth. Further research is needed to corroborate these findings and shed light on the biological mechanisms by which these types of chemicals may influence the secondary sex ratio.

## Background

Polybrominated biphenyl (PBB), a brominated flame retardant, was used in the United States in the 1970's and added to commercial products such as plastics, textiles, and electronics. The manufacture of PBB was discontinued in the United States in 1976 following a large-scale contamination incident. In 1973, a company that manufactured two products (FireMaster, a fire retardant mixture of PBBs and NutriMaster, a feed-grade magnesium oxide supplement for cattle) inadvertently delivered FireMaster to Michigan Farm Bureau Services where it was mixed into animal feed that was shipped to feed mills across the state. Between 1973 and 1974, the PBB-contaminated feed was ingested by animals, and ultimately by Michigan residents through meat, milk, eggs and other animal products. Most Michigan residents had low but detectable concentrations of PBB in their serum; however, high PBB concentrations were detected in families residing on quarantined farms which received the contaminated feed and in neighboring families who purchased food from these farms. Nearly 4,000 of these individuals were enrolled in a cohort study established in 1976 by the Michigan Department of Public (now Community) Health to track the long-term health effects of PBB exposure [1]. This cohort has been followed prospectively since that time, and by design includes information linking family members. Details of the incident and earlier studies have been described elsewhere [2-4]. Additionally, a number of studies have been conducted in this cohort investigating associations between PBB and PCB exposure and reproductive health outcomes [5-10].

PBBs belong to a class of structurally similar chemicals known as polyhalogenated aromatic hydrocarbons, which includes other potential endocrine disruptors such as dioxins, furans, and polychlorinated biphenyls (PCBs). PCBs were manufactured in the United States from the 1930's to 1970's and used as lubricants and coolants in electrical equipment [11]. Evidence of the toxic effects of PCBs and their accumulation in the environment led to their ban in the late 1970's. Because there was concern for widespread human PCB exposure, Michigan PBB cohort members also had PCB exposure concentrations measured at enrollment into the PBB cohort; PCB concentrations were similar to that in the general population [12,13]. The primary source of exposure to PCB in the general population is through contaminated food; mostly from fish obtained from PCB-contaminated waters [11]. Although the production of PBBs and PCBs has ceased, they remain a public health concern because of their environmental persistence. The estimated half-life of PBB in humans is about 10.8 years [14], and ranges from 13–29 years in females [15]. The estimated half-life of PCBs in humans ranges from less than one to 10 years or more (reviewed in [16]). In addition, PBBs and PCBs are lipophilic and can be transferred in utero and through breast milk [17-19].

The secondary sex ratio, defined as the ratio of males to females at birth, is of interest in the scientific community. Studies have suggested that this ratio is declining in the United States and in other countries [20-23]. Generally held to be about 104 to 106 males to 100 females world-wide, a number of factors are suspected to influence the sex ratio, including maternal and paternal age, birth order, plurality, and race/ethnicity (reviewed in [21,24,25]). In addition, there is increasing evidence that exposure to environmental toxins, including endocrine disruptors may influence the sex ratio. While some epidemiological studies have reported decreases in the secondary sex ratio as a result of parental exposures, others have not. The 1976 Seveso, Italy industrial accident in which some residents were exposed to high levels of dioxins, found a significant decline in males births in couples in which the fathers were highly exposed [26]. Studies in other populations, have also reported a reduced sex ratio with parental exposure to dioxins, PCBs and related environmental pollutants [27-32]. However, some studies have suggested increases or little if any association between exposure to environmental pollutants and the secondary sex ratio [31,33-36]. The Michigan Long-Term PBB Study presents a unique opportunity to study the association of parental exposures to PBB and PCB and the sex ratio at birth of their offspring.

## Methods

### Study Population

The participants of the present study were the offspring of female PBB cohort members born during 1975–1988, potentially exposed to maternal PBB in utero. Birth records were available beginning in 1975 and birth records after 1988 were excluded because after this time there were no births in which the father was a cohort member. Earlier births (those closer to the contamination period) were more likely from parents who were both enrolled in the cohort. Most cohort members were enrolled as part of a household that lived or purchased food from a quarantined farm.

Offspring were identified by matching demographic information of cohort mothers (born before July 1973) to maternal information in the Michigan electronic birth files. These matches were verified using cohort registry records, and additional births were identified from cohort infant enrollment records. We could not obtain electronic birth records for offspring born outside Michigan so these births were excluded (n = 84). Paternal information, father's name and age were determined from a manual search of cohort registry records and checked against paper copies of the birth certificate. The studies from which these data were derived have undergone human subjects review and approval by IRBs at the Michigan Department of Community Health and Emory University and informed consent was obtained from all participants.

### Exposure assessment

The Michigan PBB cohort was predominately exposed to a mixture of PBBs that contained mostly PBB-153 (60%) [37]. PBB-153, or 2,2'4,4'5,5'-hexabromobiphenyl, was measured in serum samples collected from PBB cohort participants by the Michigan Department of Community Health Bureau of Laboratories. The serum samples were first extracted with 1:1 petroleum ether-ethyl or 1:1 hexane-ether, and then passed through either a Florisil or Florisil and silica gel column. PBBs were detected and quantitated using gas chromatography with electron capture detection. The coefficients of variation ranged from 7.1% to 14.0% [38,39] and the limit of detection (LOD) was one microgram per liter (μg/L). PCB determination was based on Aroclor 1254. The coefficients of variation ranged from 12% to 30% [38,39] and the LOD was 5 μg/L. The Bureau of Laboratories used a Double Determination method which measured both total PBB and total PCB exposure in the same serum sample, so both concentrations were available for 85% of cohort members. All serum samples were collected from non-fasting participants, and lipids were not measured.

Serum samples from most of the parents were collected in 1976–1979 when they enrolled in the PBB cohort. Maternal samples were collected on average four years after their offspring's birth (range: three years before to 12 years after offspring's birth) and paternal samples were collected on average one year after their offspring's birth (range: three years before to 11 years after offspring's birth). Because of these varying times from the parents' blood collection to the offspring's birth, we estimated maternal and paternal PBB at the time of conception of the offspring based on a one-compartment first-order mixed-effects decay model [40]. For the estimated maternal PBB, we calculated a decay estimate (λ) using the parameters specified in the decay model described in Terrell et al. [40], which includes the mother's age at exposure to PBB, body mass index (BMI), smoking history, parity, and breast-feeding history. We then calculated the estimated PBB based on the formula [estimated PBB = enrollment PBB × exp (λt)], where (t) is the time between the offspring's conception date and the date when the mother's serum sample was collected. Likewise, we developed a decay model for paternal PBB exposure using a similar methodology as described in Terrell et al. [40]. The estimated paternal PBB was calculated using the above formula. The decay estimate (λ) was based on the father's age at exposure to PBB and BMI and (t) was the time between the offspring's conception date and the date when the father's serum sample was collected. Because some parents in the present study did not have their enrollment PBB concentration measured before the birth of their offspring, the decay model extrapolated backwards for those offspring born before their parent's PBB concentrations were measured. Because this cohort had relatively low serum PCB concentrations, we used the parents' PCB level collected at the PBB study enrollment period as the estimate of PCB exposure at the time of the offspring's conception.

### Statistical data analysis

Information from the electronic birth file used in this study included: offspring's sex, mother's age at offspring's birth (as a continuous variable and in categories split at the 90^th ^percentile of <30 and ≥ 30 years), father's age at offspring's birth (as a continuous variable and in categories split at the 90^th ^percentile of <35 and ≥ 35 years), mother's education at offspring's birth (≤HS and >HS), father's education at offspring's birth (≤HS and >HS), birth order (first-born and non first-born), plurality (for exclusion of multiple births), gestational age (for calculation of conception date and as a covariate), and father's race (for exclusion of non-white fathers). The population of the Long-Term study was 98% white, so we excluded offspring if the father's race was listed as non-white or missing on the birth record.

Parental information obtained from historic records of the Long-Term PBB Study included: earliest (enrollment) serum PBB and PCB exposure measurements and height and weight at enrollment to calculate body mass index (BMI). Analyses that included BMI were restricted to females at least 16 years old at enrollment in the cohort and males at least 19 years old at enrollment in the cohort, accounting for later growth spurts that often occur in males. BMI was categorized based on standard classifications from CDC of under (< 18.5 kg/m^2^), normal (18.5–24.9 kg/m^2^) and overweight (≥ 25 kg/m^2^) and also as a two-level variable for overweight versus normal and underweight combined.

Additional maternal information was obtained from structured telephone interviews conducted with female cohort members during 1997–1998 and 2003–2006 which collected detailed reproductive, hormonal, and lifestyle information. From the telephone interviews we obtained data on months of unprotected intercourse to achieve the pregnancy (1–3 months and >3 months) and when not available in the electronic birth file, information on offspring's gestational age and birth order.

For the exposure variables, PBB and PCB concentrations were modeled as: two groups, split at the median concentrations (medians: 3 μg/L for maternal enrollment PBB, 2 μg/L for maternal estimated PBB, 6 μg/L for paternal enrollment and estimated PBB, 6 μg/L for maternal PCB, and 8 μg/L for paternal PCB); and as continuous variables, log-transformed because of their skewed distributions. In order to evaluate a possible dose response, we also categorized enrollment PBB and estimated PBB concentrations into three groups based on the limit of detection and the median concentration of those above. The categories were (low: ≤1, moderate: >1–<4, high: ≥4 μg/L) for maternal PBB and (low: ≤1, moderate: >1–<6, high: ≥6 μg/L) for paternal PBB.

We were interested in modeling the probability of a male birth in relation to parents' PBB or PCB exposure concentrations. We used logistic regression analyses to model the odds of a male birth and calculated unadjusted odds ratios, adjusted odds ratios (AOR) and 95% confidence intervals (CI). Because the Long-Term study was, by design a cohort of families, our study population of offspring included up to five siblings. Therefore, all analyses were performed using generalized estimating equations (GEE) to account for the lack of independence between offspring from the same family (link = logit; covariance structure = exchangeable). To assess potential confounding, we explored the unadjusted associations between each covariate and the outcome, and each covariate with the exposure variables (cut-off p < 0.10). In multivariate analyses, we ran a series of models with potential confounders and maternal or paternal enrollment PBB, estimated PBB at conception or enrollment PCB exposure. Year of offspring's birth was included as a covariate in all adjusted models. Covariates were removed sequentially using backward elimination and were retained if the main exposure odds ratios changed by at least ten percent. We examined models where maternal and paternal exposure were modeled separately and combined. However, because maternal and paternal serum concentrations were correlated, we did not include them in a model simultaneously. Finally, to examine combined parents' exposure (interaction term), we considered only three categories: where both parents had exposure < the median concentrations (referent group), where both parents had exposure ≥ the median concentrations, and the combination of parents' with discordant exposure levels. All analyses were performed using SAS v9.2 [41].

## Results

### Population characteristics

In total, we identified 922 Michigan born offspring to 496 PBB cohort mothers from linkage with electronic birth records. For 366 of these offspring, we identified 208 fathers who were also participants in the PBB cohort. We excluded offspring from the study for the following reasons: no maternal PBB measurement (n = 33); father's race missing or listed as non-white on the offspring's birth record (n = 8); and non-singleton births (n = 16). Thus, our final sample included 865 Michigan born offspring to 479 PBB cohort mothers. Of these, 300 offspring had mothers and fathers who were both in the cohort (n = 171 pairs of mothers and fathers). The overall proportion male among the 865 offspring was 0.542 (corresponding sex ratio = 1.18). The proportion male among these offspring was slightly higher than the national male proportion [21] of 0.514 (binomial test: p = 0.10).

### PBB and PCB concentrations

**Figure 1:**
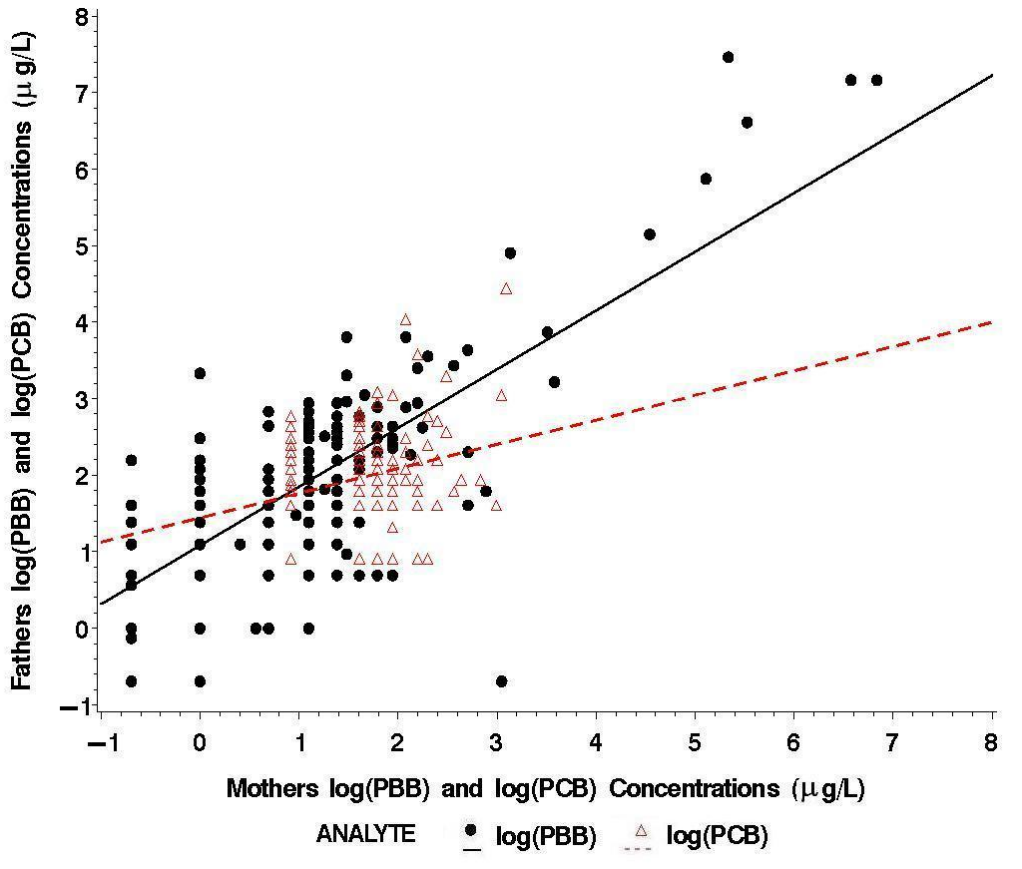
**Relationship between parents' serum log-transformed PBB and log-transformed PCB concentrations (N = 300 offspring)**. Spearman correlation coefficients: PBB, r_s _= 0.64; PCB, r_s _= 0.19.

The mean age of mothers during the PBB exposure period (based on age in 1973) was 17 years (range: infancy-38 years). Fathers' mean age during the PBB exposure period was 25 years (range: 13–61 years). In general, fathers had higher PBB and PCB concentrations than the mothers (maternal PBB range: < LOD-933 μg/L; 20% < LOD; paternal PBB range: < LOD-1744 μg/L; 5% < LOD; maternal PCB range: < LOD-78 μg/L; 43% < LOD; paternal PCB range: < LOD-85 μg/L; 17% < LOD). There were positive, although weak correlations between mothers' log-transformed PBB and PCB concentrations (n = 434 mothers, r_s _= 0.14, p = 0.004) and between fathers' log-transformed PBB and PCB concentrations (n = 162 fathers, r_s _= 0.13, p = 0.10). As shown in Figure 1, serum PBB concentrations were much higher than serum PCB concentrations. In addition, there was a significant positive relationship between mothers' and fathers' log-transformed exposure concentrations for PBB (r_s _= 0.64, p < 0.001) and PCB (r_s _= 0.19, p = 0.002).

### Association with sex ratio

Table 1 gives results from unadjusted GEE models examining the odds of a male birth among offspring whose mothers' were in the cohort by potential confounding variables (n = 865 offspring, n = 479 mothers). Although the results for paternal BMI and offspring gestational age were imprecise, increased odds of a male birth were seen for offspring born to fathers with high paternal BMI at enrollment (BMI ≥ 25 kg/m^2^) compared to fathers with normal paternal BMI at enrollment (18.5–24.9 kg/m^2^) (OR = 1.43, 95% CI: 0.98–2.09) and for offspring born before 37 weeks gestation than offspring born 37 to < 42 weeks gestation (OR = 1.78, 95% CI: 0.86–3.67). The other covariates, maternal or paternal age during the PBB exposure period, maternal or paternal age at offspring's birth, maternal BMI at enrollment, maternal or paternal education at offspring's birth, months of unprotected intercourse to achieve the pregnancy and birth order showed little association with the odds of a male birth.

**Table 1:** Characteristics of Michigan births and unadjusted odds ratios for a male birth from parents in the Michigan Long-Term PBB Study (n = 865 offspring born 1975–1988)

Characteristic	N% Offspring	Unadjusted OR^ (95% CI)
Maternal age at offspring's birth (years)		

< 30	735 (85)	1.00

≥ 30	130 (15)	1.29 (0.89, 1.86)

		

Paternal age at offspring's birth (years)		

< 35	740 (89)	1.00

≥ 35	89 (11)	0.95 (0.64, 1.39)

		

Maternal education at offspring's birth		

≤ HS	543 (63)	1.00

> HS	320 (37)	1.09 (0.83, 1.42)

		

Paternal education at offspring's birth		

≤ HS	524 (63)	1.00

> HS	304 (37)	1.06 (0.81, 1.38)

		

Maternal BMI at enrollment (kg/m^2^)*		

< 18.5	60 (9)	0.93 (0.55, 1.58)

18.5–24.9	466 (67)	1.00

≥ 25	171 (24)	0.96 (0.72, 1.29)

		

Paternal BMI at enrollment (kg/m^2^)*		

< 18.5	4 (1)	0.77 (0.27, 2.23)

18.5–24.9	173 (57)	1.00

≥ 25	129 (42)	1.43 (0.98, 2.09)

		

Months unprotected intercourse to achieve pregnancy **		

1–3 months	300 (59)	1.00

>3 months	210 (41)	0.94 (0.67, 1.31)

		

Birth order		

First-born	325 (38)	1.00

Non first-born	540 (62)	1.08 (0.81, 1.42)

		

Offspring gestation (weeks)		

< 37	30 (4)	1.78 (0.86, 3.67)

37 to <42	664 (77)	1.00

≥ 42	165 (19)	0.99 (0.71, 1.37)

^ ORs are unadjusted for covariates, but adjusted for offspring born to the same mother or father

* Maternal enrollment BMI restricted to mothers' ages ≥ 16 years and fathers' ages ≥ 19 years

** Mothers who reported "doing something to prevent the pregnancy" were not asked the follow-up question of how many months of unprotected intercourse it took to achieve pregnancy (for n = 112 offspring)

We present the crude (unadjusted for offspring born to the same mother or father), unadjusted and adjusted GEE models for the odds of a male birth by parents' enrollment PBB exposure in Table 2.

**Table 2:** Odds ratios (OR) for a male birth among offspring from parents in the Michigan Long-Term PBB Study with enrollment serum PBB concentrations

		N (%)	Crude^	Unadjusted *	Adjusted **
Exposure Variables (μg/L)		Offspring	OR (95% CI)	OR (95% CI)	OR (95% CI)

Model 1 (Maternal enrollment PBB only):					

Maternal PBB		865			

< 3		477 (55)	1.00	1.00	1.00

≥ 3		388 (45)	0.92 (0.70, 1.21)	0.93 (0.72, 1.20)	0.93 (0.72, 1.20)

Model 2 (Paternal enrollment PBB only):					

Paternal PBB		300			

< 6		160 (53)	1.00	1.00	1.00

≥ 6		140 (47)	1.11 (0.70, 1.75)	0.95 (0.65, 1.39)	1.18 (0.76, 1.83)

Model 3 (Maternal and Paternal enrollment PBB combined):					

Maternal PBB	Paternal PBB	300			

< 3	< 6	120 (40)	1.00	1.00	1.00

other combinations		70 (23)	0.99 (0.55, 1.79)	0.84 (0.52, 1.37)	0.92 (0.61, 1.40)

≥ 3	≥ 6	110 (37)	1.35 (0.80, 2.28)	1.14 (0.73, 1.79)	1.43 (0.89, 2.29)

^ Crude ORs are unadjusted for covariates or offspring born to the same mother or father

* ORs are unadjusted for covariates, but adjusted for offspring born to the same mother or father

** AORs are adjusted for covariates (Model 1 adjusted for year of offspring's birth; Models 2 and 3 adjusted for year of offspring's birth and paternal BMI as low and normal vs. high) and adjusted for offspring born to the same mother or father

In the model with only maternal PBB exposure (Model 1), there was little effect of exposure on the odds of a male birth for offspring born to mothers with PBB ≥ 3 μg/L compared to mothers with PBB < 3 μg/L (n = 865 offspring, n = 479 mothers; AOR = 0.93, 95% CI: 0.72, 1.20). There was a small increase in the odds of a male birth in the model with only paternal PBB exposure (Model 2, n = 300 offspring, n = 171 fathers) with an adjusted odds ratio of 1.18 (95% CI: 0.76, 1.83) for offspring born to fathers with PBB ≥ 6 μg/L compared to fathers with PBB < 6 μg/L. When we considered the model with combined maternal and paternal PBB exposure, the odds ratio for offspring where both parents had PBB exposure ≥ the median concentrations (≥ 3 μg/L for mothers and ≥ 6 μg/L for fathers) compared to where both parents had PBB exposure < the median concentrations was increased, but imprecise (Model 3, n = 300 offspring, n = 171 pairs of mothers and fathers). The adjusted odds ratio was 1.43 (95% CI: 0.89, 2.29), when adjusted for paternal BMI and year of offspring's birth.

Table 3 shows the results when we modeled the odds of a male birth by estimated PBB at the conception date of the offspring. The odds ratios in models 1–3 with estimated PBB were similar to those with enrollment PBB in Table 2. In model 3 (n = 226 offspring, 131 pairs of mothers and fathers), the adjusted odds ratio of a male birth for parents with estimated PBB exposure ≥ the median concentrations (≥ 2 μg/L for mothers and ≥ 6 μg/L for fathers) compared to parents with estimated PBB exposure < the median concentrations was attenuated (AOR = 1.40, 95% CI: 0.84, 2.35).

**Table 3:** Odds ratios (OR) for a male birth among offspring from parents in the Michigan Long-Term PBB Study with estimated serum PBB concentrations

		N (%)	Crude^	Unadjusted *	Adjusted**
Exposure Variables (μg/L)		Offspring	OR (95% CI)	OR (95% CI)	OR (95% CI)

Model 1 (Maternal estimated PBB only):					

Maternal PBB		681			

< 2		354 (52)	1.00	1.00	1.00

≥ 2		327 (48)	0.97 (0.72, 1.32)	0.97 (0.73, 1.29)	1.00 (0.75, 1.33)

Model 2 (Paternal estimated PBB only):					

Paternal PBB		297			

< 6		167 (56)	1.00	1.00	1.00

≥ 6		130 (44)	1.05 (0.67, 1.67)	0.90 (0.61, 1.32)	1.08 (0.71, 1.66)

Model 3 (Maternal and Paternal estimated PBB combined):					

Maternal PBB	Paternal PBB	226			

< 2	< 6	83 (37)	1.00	1.00	1.00

other combinations		65 (29)	0.87 (0.45, 1.67)	0.89 (0.50, 1.59)	0.87 (0.52, 1.46)

≥ 2	≥ 6	78 (34)	1.21 (0.65, 2.27)	1.13 (0.67, 1.90)	1.40 (0.84, 2.35)

^ Crude ORs are unadjusted for covariates or offspring born to the same mother or father

* ORs are unadjusted for covariates, but adjusted for offspring born to the same mother or father

** AORs are adjusted for covariates (Model 1 adjusted for year of offspring's birth; Models 2 and 3 adjusted for year of offspring's birth and paternal BMI as low and normal vs. high) and adjusted for offspring born to the same mother or father

We examined the odds of a male birth by parents' enrollment PCB exposure in Table 4. In the model with only maternal PCB exposure (Model 1), there was no effect of maternal PCB exposure on the odds of a male birth. However, in the model with only paternal PCB exposure (Model 2), there was a non-significant increase in the odds of a male birth for offspring born to fathers with PCB ≥ 8 μg/L (n = 253 offspring, n = 144 fathers, AOR = 1.24, 95% CI: 0.81, 1.88; referent group: PCB < 8 μg/L). Increased odds of a male birth was also seen in the model with combined parents' PCB exposure (Model 3, n = 253 offspring, n = 144 pairs of mothers and fathers). The adjusted odds ratio was 1.53 (95% CI: 0.93, 2.52) for offspring born to parents with PCB exposure ≥ the median concentrations (≥ 6 μg/L for mothers and ≥ 8 μg/L for fathers) compared to parents with PCB exposure < the median concentrations; however, the odds ratio was imprecise.

**Table 4:** Odds ratios (OR) for a male birth among offspring from parents in the Michigan Long-Term PBB Study with enrollment serum PCB concentrations

		N (%)	Crude^	Unadjusted *	Adjusted**
Exposure Variables (μg/L)		Offspring	OR (95% CI)	OR (95% CI)	OR (95% CI)

Model 1 (Maternal PCB only):					

Maternal PCB		790			

< 6		444 (56)	1.00	1.00	1.00

≥ 6		346 (44)	1.02 (0.77, 1.35)	0.99 (0.76, 1.30)	1.01 (0.77, 1.32)

Model 2 (Paternal PCB only):					

Paternal PCB		253			

< 8		141 (56)	1.00	1.00	1.00

≥ 8		112 (44)	1.06 (0.64, 1.75)	1.23 (0.83, 1.82)	1.24 (0.81, 1.88)

Model 3 (Maternal and Paternal PCB combined):					

Maternal PCB	Paternal PCB	253			

< 6	< 8	80 (32)	1.00	1.00	1.00

other combinations		116 (46)	1.26 (0.71, 2.22)	1.23 (0.77, 1.97)	1.00 (0.63, 1.57)

≥ 6	≥ 8	57 (22)	1.13 (0.57, 2.24)	1.39 (0.81, 2.40)	1.53 (0.93, 2.52)

^ Crude ORs are unadjusted for covariates or offspring born to the same mother or father

* ORs are unadjusted for covariates, but adjusted for offspring born to the same mother or father

** AORs are adjusted for covariates (Model 1 adjusted for year of offspring's birth; Models 2 and 3 adjusted for year of offspring's birth and paternal BMI as low and normal vs. high) and adjusted for offspring born to the same mother or father

When we examined PBB and PCB exposure as continuous log-transformed variables, the results were consistent with those presented in Tables 2, 3 and 4. For the adjusted maternal PBB only model the AOR = 1.00 (95% CI: 0.80, 1.25). For the paternal PBB only model, there was a 15% increase in the odds of a male birth for a 10 μg/L increase in the natural log of paternal PBB concentration (AOR = 1.15, 95% CI: 0. 80, 1.65). In the combined maternal and paternal PBB model, there was a 6% increase in the odds of a male birth (AOR = 1.06, 95% CI: 0.97, 1.17) for a 10 μg/L increase in the natural log of maternal and paternal PBB concentrations. For PCB exposure, the odds of a male birth for a 10 μg/L increase in the natural log of serum PCB concentrations were as follows: in the maternal PCB only model, a 25% increase (AOR = 1.25, 95% CI: 0.79, 1.98); in the paternal PCB only model, a 34% increase (AOR = 1.34, 95% CI: 0.60, 2.98); and in the combined maternal and paternal PCB model, a 13% increase (AOR = 1.13, 95% CI: 0.85, 1.49).

We also examined enrollment PBB exposure categorized as three groups. In the model with maternal enrollment PBB only (n = 865 offspring, n = 479 mothers), there was a non-significant increase in the odds of a male birth for offspring born to mothers with moderate PBB exposure (PBB > 1–< 4 μg/L, n = 268 offspring, AOR = 1.22, 95% CI: 0.89, 1.67) compared to mothers with low PBB exposure (PBB ≤ 1 μg/L, n = 308 offspring). This was less evident for mothers with high PBB exposure compared to mothers with low PBB exposure (PBB ≥ 4 μg/L, n = 289 offspring, AOR = 1.05, 95% CI: 0.77, 1.43). In the model with paternal enrollment PBB only (n = 300 offspring, n = 171 fathers), we found increased odds of a male birth for offspring born to fathers with moderate PBB exposure (PBB > 1–< 6 μg/L, n = 135 offspring, AOR = 1.53, 95% CI: 0.81, 2.89) and high PBB exposure (PBB ≥ 6 μg/L, n = 140 offspring, AOR = 1.69, 95% CI: 0.85, 3.34) when either were compared to fathers with low PBB exposure (PBB ≤ 1 μg/L, n = 25 offspring). In the combined maternal and paternal enrollment PBB model (n = 300 offspring, n = 171 mothers and fathers), we considered where both parents had high PBB exposure (maternal PBB ≥ 4 μg/L and paternal PBB ≥ 6 μg/L, n = 81 offspring) and compared this to the referent group where both parents had low PBB exposure (maternal PBB < 4 μg/L and paternal PBB < 6 μg/L, n = 19 offspring). We found increased odds of a male birth when both parents had high PBB exposure after adjusting for paternal BMI and year of offspring's birth (AOR = 2.56, 95% CI: 1.32, 4.98). Finally, in the model with all other combinations of parents' PBB exposure (where parents' had discordant exposure levels, n = 200 offspring) compared to the referent group of parents with low PBB exposure (n = 19 offspring) the odds ratio was in the same direction, but attenuated (AOR = 1.24, 95% CI: 0.64, 2.38). The models with estimated PBB exposure categorized as three groups had similar results.

To verify our findings presented above, we performed two additional analyses. First, because the decay model was used to estimate PBB exposure backwards for offspring born before their parents exposure was collected, we repeated the adjusted models in Table 3 excluding those offspring (n = 116 offspring born before their mothers' PBB measurement; n = 118 offspring born before their fathers' PBB measurement). For the maternal estimated PBB only model (n = 565 offspring, 340 mothers), the results did not change from those in Table 3, Model 1 (maternal estimated PBB ≥ 2 μg/L: AOR = 0.99, 95% CI: 0.72–1.37; referent group: maternal estimated PBB < 2 μg/L). For the paternal estimated PBB only model (n = 179 offspring, 118 fathers), the adjusted odds ratio was larger than in Table 3, Model 2 (paternal estimated PBB ≥ 6 μg/L: AOR = 1.63, 95% CI: 0.94–2.83; referent group: paternal estimated PBB < 6 μg/L). Likewise, for the combined maternal and paternal estimated PBB model (n = 138 offspring, n = 98 pairs of mothers and fathers), the adjusted odds ratio was increased but had wide confidence intervals due to small numbers (maternal estimated PBB ≥ 2 μg/L and paternal estimated PBB ≥ 6 μg/L, n = 43 offspring: AOR = 2.47, 95% CI: 1.15–5.28; referent group: maternal estimated PBB < 2 μg/L and paternal estimated PBB < 6 μg/L, n = 56 offspring).

Second, because changes in BMI or pregnancy and breastfeeding history may affect mothers' PBB or PCB concentrations, we restricted the enrollment maternal models to include only the first offspring born after the mothers' enrollment PBB or PCB concentration was measured. For the maternal enrollment PBB only model (n = 407 offspring), the adjusted odds ratio was comparable to Table 2, Model 1 (maternal PBB ≥ 3 μg/L: AOR = 0.91, 95% CI: 0.61–1.35; referent group: maternal PBB < 3 μg/L). For the maternal enrollment PCB only model (n = 346 offspring), the odds ratio was 1.39 (95% CI: 0.89–2.17), for offspring born to mothers with PCB ≥ 6 μg/L compared to mothers with PCB < 6 μg/L.

## Discussion

Among this Michigan cohort of 865 offspring with potential in utero PBB exposure, the overall proportion of male births was 0.542. This was higher than the national male proportion of 0.514 [21] and higher than that of Michigan births over the same time period (range: 0.511–0.516, Source: 1975–1988 Live Birth Files, Vital Records and Health Data Development Section, MDCH). When we considered the subset of births where both parents were in the cohort, there was a suggestion of increased odds of a male birth among offspring born to where both parents had PBB exposure ≥ the median concentrations. The results were consistent when both parents had PCB exposure ≥ the median concentrations. When PBB exposure was categorized into three groups, we found a statistically significant increase in the odds of a male birth where both parents had high PBB exposure compared to where both parents had low PBB exposure, but the referent group was based on small numbers and the confidence interval became wide. In models where only one parent's exposure was considered, there was a suggestion of increased odds of a male birth for paternal PBB or PCB exposure only, but not for maternal PBB or PCB exposure only.

To our knowledge, this is the first study to investigate the relationship of the secondary sex ratio and PBB exposure. Therefore, comparison of our results is limited to studies that have measured other polyhalogenated aromatic hydrocarbons, such as PCBs, dioxins, and furans. A few studies that considered PCB exposure found increases in the sex ratio [31,35,36], which is similar to our findings. Further, several studies have found associations with fathers exposure but not necessarily mothers exposure in relation to the sex ratio (reviewed in [24]). Our results were not consistent, however, with other studies that found a decrease in the secondary sex ratio with parental exposure to related chemicals [26-32].

The biological mechanism by which exposure to PBBs may influence sex ratio remains unclear. The main congener in the mixture of PBBs to which the Michigan residents were exposed was PBB-153, which has been shown to exhibit estrogenic, anti-estrogenic, or anti-androgenic activity, similarly as for some PCB congeners [42,43]. Additionally, there is some evidence to suggest that possible alterations in sex ratio may be influenced by parental hormone levels around the time of conception [44]. However, it is unknown if PBBs mediate changes in parental hormone levels that would increase the odds of a male birth, as seen in our study. On the paternal side, whether exposure to these types of chemicals causes the preferential survival of Y sperm over X sperm has been considered as a possible mechanism; although the findings in studies have been inconsistent [45,46]. On the maternal side, it is unknown whether exposure to these types of chemical could cause an increase in early loss of XX embryos.

Our sample included 865 offspring born to mothers in the cohort during 1975–1988, but only 300 offspring born to fathers in the cohort. This may have biased our results because mothers and fathers who were both in the cohort had higher serum PBB concentrations compared to where only the mother was in the cohort but not the father. In addition, we excluded birth records for offspring born to cohort mothers after 1988. When we considered a model with only maternal enrollment PBB for all available data (n = 1392 offspring; n = 865 born 1975–1988; n = 527 born 1989–2005), there remained no effect of maternal exposure on the odds of a male birth (maternal PBB ≥ 3 μg/L: AOR = 1.04, 95% CI: 0.85–1.28; referent group: maternal PBB < 3 μg/L). Ultimately, we excluded these later offspring from our study sample because we could not effectively account for any secular trends in sex ratio, which may have affected the later births. In addition, the estimated maternal PBB at conception would have been based on maternal enrollment PBB collected more than 13 years before these offspring were born. We obtained the sex of offspring from electronic birth records, but we could not obtain records for out of state births. It is unlikely that this was a source of bias, because the proportion male birth among the 84 out of state births (proportion male = 0.548) was not different to that of our final sample (proportion male = 0.542). Among the 33 offspring where the mother did not have a PBB measurement, the proportion male birth was slightly less (proportion male = 0.485). Based on the results of our study, paternal PBB exposure had a greater effect than maternal PBB exposure on the odds of a male birth. However, determining whether either parent's exposure separately or their combined exposure would contribute to a skewed sex ratio was complicated because exposure levels from parents in the same family were correlated.

We considered several covariates and their association with the odds of a male birth. We found increased odds of a male birth for high paternal BMI (BMI ≥ 25 kg/m^2^), but not for high maternal BMI. In this population, BMI has been shown to slow the decay of PBB in the body [15,40]. Therefore, as a confounder, we retained paternal BMI in models where paternal exposure was considered. Although, we cannot rule out the potential for bias, given that weight and height were self-reported by participants. It is possible that the heavier fathers at enrollment into the cohort had higher concentrations of PBB at the time of their offspring's conception. Maternal weight or BMI has been considered in other sex ratio studies [31,47], but to our knowledge this is the first study to consider paternal BMI in relation to the odds of a male birth. As expected, for gestational age we found increased odds of a male birth for offspring born prior to 37 weeks gestation. However, we have previously found no association between gestational age and PBB exposure in this population [8,9], and thus gestational age was not a confounder in our analyses. We did not find an association with other factors that are reported to influence the secondary sex ratio, such as maternal and paternal age or birth order.

## Conclusion

Our results add to the body of literature on the possible effects of environmental pollutants on the secondary sex ratio. This study includes a well-defined period of PBB exposure, and over 30 years of birth record and cohort registry data from the Long-Term study. In this population, paternal exposure alone and combined maternal and paternal exposure (for PBB or PCB) increased the odds of a male birth. Further research is needed to corroborate these findings and shed light on the biological mechanisms by which these types of chemicals may influence the secondary sex ratio.

## Abbreviations

PBB: polybrominated biphenyl; PCB: polychlorinated biphenyl; BMI: body mass index; μg/L: microgram per liter; LOD: limit of detection; OR: odds ratio; CI: confidence interval; AOR: adjusted odds ratio.

## Competing interests

The authors declare that they have no competing interests.

## Authors' contributions

All authors have made substantial contribution to this study and to the writing and editing of this manuscript. Additional contributions are as follows: MM, LC and CS designed the study and provided historical cohort data; JW retrieved and matched cohort records and verified offspring/parental relationships; MT and AB performed statistical analyses. All authors read and approved the final manuscript.

## References

[B1] Landrigan PJ Wilcox KR Jr Silva J Jr Humphrey HE Kauffman C Heath CW Jr Cohort study of Michigan residents exposed to polybrominated biphenyls: epidemiologic and immunologic findings Ann N Y Acad Sci 1979 320 284 294 222186 10.1111/j.1749-6632.1979.tb56611.x

[B2] Fries GF The PBB episode in Michigan: an overall appraisal Crit Rev Toxicol 1985 16 105 156 10.3109/10408448509056268 3002722

[B3] Humphrey H Hayner N Polybrominated Biphenyls: An agricultural incident and its consequences: An epidemiological investigation of human exposure Trace Subst Environ Health 1976 9 57 63

[B4] Kay K Polybrominated biphenyls (PBB) environmental contamination in Michigan, 1973–1976 Environ Res 1977 13 74 93 10.1016/0013-9351(77)90006-8 191251

[B5] Blanck HM Marcus M Tolbert PE Rubin C Henderson AK Hertzberg VS Zhang RH Cameron L Age at menarche and tanner stage in girls exposed in utero and postnatally to polybrominated biphenyl Epidemiology 2000 11 641 647 10.1097/00001648-200011000-00005 11055623

[B6] Davis SI Blanck HM Hertzberg VS Tolbert PE Rubin C Cameron LL Henderson AK Marcus M Menstrual function among women exposed to polybrominated biphenyls: a follow-up prevalence study Environ Health 2005 4 15 10.1186/1476-069X-4-15 16091135 PMC1201158

[B7] Small CM Cheslack-Postava K Terrell M Blanck HM Tolbert P Rubin C Henderson A Marcus M Risk of spontaneous abortion among women exposed to polybrominated biphenyls Environ Res 2007 105 247 255 10.1016/j.envres.2006.11.010 17239850 PMC2237897

[B8] Sweeney AM Symanski E The influence of age at exposure to PBBs on birth outcomes [see comment] Environ Res 2007 105 370 379 10.1016/j.envres.2007.03.006 17485077

[B9] Givens ML Small CM Terrell ML Cameron LL Michels Blanck H Tolbert PE Rubin C Henderson AK Marcus M Maternal exposure to polybrominated and polychlorinated biphenyls: Infant birth weight and gestational age Chemosphere 2007 69 1295 1304 10.1016/j.chemosphere.2007.05.031 17617441 PMC2075473

[B10] Hoffman CS Small CM Blanck HM Tolbert P Rubin C Marcus M Endometriosis among women exposed to polybrominated biphenyls Ann Epidemiol 2007 17 503 510 10.1016/j.annepidem.2006.11.005 17448678 PMC2075471

[B11] Agency for Toxic Substances and Disease Registry (ATSDR) Toxicological profile for polychlorinated biphenyls (PCBs) 2000 Atlanta, GA: U.S. Department of Health and Human Services, Public Health Service 36888731

[B12] Kreiss K Roberts C Humphrey HE Serial PBB levels, PCB levels, and clinical chemistries in Michigan's PBB cohort Arch Environ Health 1982 37 141 147 6284069 10.1080/00039896.1982.10667553

[B13] Kreiss K Studies on populations exposed to polychlorinated biphenyls Environ Health Perspect 1985 60 193 199 10.2307/3429961 3928345 PMC1568576

[B14] Rosen DH Flanders WD Friede A Humphrey HE Sinks TH Half-life of polybrominated biphenyl in human sera Environ Health Perspect 1995 103 272 274 10.2307/3432548 7768229 PMC1519084

[B15] Blanck HM Marcus M Hertzberg V Tolbert PE Rubin C Henderson AK Zhang RH Determinants of polybrominated biphenyl serum decay among women in the Michigan PBB cohort Environ Health Perspect 2000 108 147 152 10.2307/3454513 10656855 PMC1637888

[B16] Shirai JH Kissel JC Uncertainty in estimated half-lives of PCBS in humans: impact on exposure assessment Sci Total Environ 1996 187 199 210 10.1016/0048-9697(96)05142-X 8711465

[B17] Eyster JT Humphrey HE Kimbrough RD Partitioning of polybrominated biphenyls (PBBs) in serum, adipose tissue, breast milk, placenta, cord blood, biliary fluid, and feces Arch Environ Health 1983 38 47 53 6299210 10.1080/00039896.1983.10543978

[B18] Miceli JN Nolan DC Marks B Hariharan M Persistence of polybrominated biphenyls (PBB) in human post-mortem tissue Environ Health Perspect 1985 60 399 403 10.2307/3429987 2992925 PMC1568566

[B19] Jacobson JL Fein GG Jacobson SW Schwartz PM Dowler JK The transfer of polychlorinated biphenyls (PCBs) and polybrominated biphenyls (PBBs) across the human placenta and into maternal milk Am J Public Health 1984 74 378 379 10.2105/AJPH.74.4.378 6322600 PMC1651484

[B20] Marcus M Kiely J Xu F McGeehin M Jackson R Sinks T Changing sex ratio in the United States, 1969–1995 Fertil Steril 1998 70 270 273 10.1016/S0015-0282(98)00149-6 9696219

[B21] Mathews TJ Hamilton BE Trend analysis of the sex ratio at birth in the United States Natl Vital Stat Rep. 2005 53 20 1 17 15974501

[B22] Moller H Change in male:female ratio among newborn infants in Denmark [see comment][comment] Lancet 1996 348 828 829 10.1016/S0140-6736(05)65253-1 8814010

[B23] Allan BB Brant R Seidel JE Jarrell JF Declining sex ratios in Canada [see comment][erratum appears in Can Med Assoc J 1997 Feb 1;156(3):348] CMAJ. 1997 156 1 37 41 9006562 PMC1226854

[B24] James WH Offspring sex ratios at birth as markers of paternal endocrine disruption Environ Res 2006 100 77 85 10.1016/j.envres.2005.03.001 15922323

[B25] Cordier S Evidence for a role of paternal exposures in developmental toxicity Basic Clin Pharmacol Toxicol 2008 102 176 181 18226072 10.1111/j.1742-7843.2007.00162.x

[B26] Mocarelli P Gerthoux PM Ferrari E Patterson DG Jr Kieszak SM Brambilla P Vincoli N Signorini S Tramacere P Carreri V Sampson EJ Turner WE Needham LL Paternal concentrations of dioxin and sex ratio of offspring Lancet 2000 355 1858 1863 10.1016/S0140-6736(00)02290-X 10866441

[B27] Hertz-Picciotto I Jusko TA Willman EJ Baker RJ Keller JA Teplin SW Charles MJ A cohort study of in utero polychlorinated biphenyl (PCB) exposures in relation to secondary sex ratio Environ Health 2008 7 37 10.1186/1476-069X-7-37 18627595 PMC2483969

[B28] Weisskopf M Anderson HA Hanrahan LP Great Lakes Consortium Decreased sex ratio following maternal exposure to polychlorinated biphenyls from contaminated Great Lakes sport-caught fish: a retrospective cohort study Environ Health. 2003 2 1 2 12694628 10.1186/1476-069X-2-2 PMC153540

[B29] Ryan JJ Amirova Z Carrier G Sex ratios of children of Russian pesticide producers exposed to dioxin Environ Health Perspect 2002 110 A699 701 12417498 10.1289/ehp.021100699 PMC1241090

[B30] Mackenzie CA Lockridge A Keith M Declining sex ratio in a first nation community Environ Health Perspect 2005 113 1295 1298 16203237 10.1289/ehp.8479 PMC1281269

[B31] Taylor KC Jackson LW Lynch CD Kostyniak PJ Buck Louis GM Preconception maternal polychlorinated biphenyl concentrations and the secondary sex ratio Environ Res 2007 103 99 105 10.1016/j.envres.2006.04.009 16780830

[B32] del Rio Gomez I Marshall T Tsai P Shao YS Guo YL Number of boys born to men exposed to polychlorinated byphenyls Lancet 2002 360 143 144 10.1016/S0140-6736(02)09386-8 12126828

[B33] Schnorr TM Lawson CC Whelan EA Dankovic DA Deddens JA Piacitelli LA Reefhuis J Sweeney MH Connally LB Fingerhut MA Spontaneous abortion, sex ratio, and paternal occupational exposure to 2,3,7,8-tetrachlorodibenzo-p-dioxin Environ Health Perspect 2001 109 1127 1132 10.2307/3454859 11712997 PMC1240473

[B34] Michalek JE Rahe AJ Boyle CA Paternal dioxin and the sex of children fathered by veterans of Operation Ranch Hand Epidemiology 1998 9 474 475 10.1097/00001648-199807000-00023 9647916

[B35] Yoshimura T Kaneko S Hayabuchi H Sex ratio in offspring of those affected by dioxin and dioxin-like compounds: the Yusho, Seveso, and Yucheng incidents Occup Environ Med 2001 58 540 541 10.1136/oem.58.8.540 11452050 PMC1740172

[B36] Karmaus W Huang S Cameron L Parental concentration of dichlorodiphenyl dichloroethene and polychlorinated biphenyls in Michigan fish eaters and sex ratio in offspring J Occup Environ Med 2002 44 8 13 10.1097/00043764-200201000-00003 11802470

[B37] Agency for Toxic Substances and Disease Registry (ATSDR) Toxicological profile for polybrominated biphenyls and polybrominated diphenyl ethers 2004 Atlanta GA: U.S. Department of Health and Human Services, Public Health Service

[B38] Burse VW Needham LL Liddle JA Bayse DD Price HA Interlaboratory comparison for results of analyses for polybrominated biphenyls in human serum J Anal Toxicol 1980 4 22 26 6098783 10.1093/jat/4.1.22

[B39] Needham LL Burse VW Price HA Temperature-programmed gas chromatographic determination of polychlorinated and polybrominated biphenyls in serum J Assoc Off Anal Chem 1981 64 1131 1137 6270054

[B40] Terrell ML Manatunga AK Small CM Cameron LL Wirth J Blanck HM Lyles RH Marcus M A Decay Model for Assessing Polybrominated Biphenyl Exposure Among Women in the Michigan Long-Term PBB Study J Expo Sci Environ Epidemiol. 2008 18 4 410 420 18183045 10.1038/sj.jes.7500633 PMC5580493

[B41] SAS Institute I SAS/STAT Software: Changes and Enhancements for Release 6.12 1996 Cary, NC.: SAS Institute, Inc

[B42] Bonefeld-Jorgensen EC Andersen HR Rasmussen TH Vinggaard AM Effect of highly bioaccumulated polychlorinated biphenyl congeners on estrogen and androgen receptor activity Toxicology 2001 158 141 153 10.1016/S0300-483X(00)00368-1 11275356

[B43] Nakari T Pessala P In vitro estrogenicity of polybrominated flame retardants Aquat Toxicol 2005 74 272 279 10.1016/j.aquatox.2005.06.004 16024102

[B44] James WH Evidence that mammalian sex ratios at birth are partially controlled by parental hormone levels at the time of conception J Theor Biol 1996 180 271 286 10.1006/jtbi.1996.0102 8776463

[B45] Tiido T Rignell-Hydbom A Jonsson BA Giwercman YL Pedersen HS Wojtyniak B Ludwicki JK Lesovoy V Zvyezday V Spano M Manicardi GC Bizzaro D Bonefeld-Jorgensen EC Toft G Bonde JP Rylander L Hagmar L Giwercman A Inuendo Impact of PCB and p,p'-DDE contaminants on human sperm Y:X chromosome ratio: studies in three European populations and the Inuit population in Greenland Environ Health Perspect 2006 114 718 724 16675426 10.1289/ehp.8668 PMC1459925

[B46] Tiido T Rignell-Hydbom A Jonsson B Giwercman YL Rylander L Hagmar L Giwercman A Exposure to persistent organochlorine polluntants associates with human sperm Y:X chromosome ratio Hum Reprod 2005 20 7 1903 9 10.1093/humrep/deh855 15860497

[B47] Cagnacci A Renzi A Arangino S Alessandrini C Volpe A Influences of maternal weight on the secondary sex ratio of human offspring Hum Reprod 2004 19 442 444 10.1093/humrep/deh071 14747194

